# Predicting task-related brain activity from resting-state brain dynamics with fMRI Transformer

**DOI:** 10.1162/imag_a_00440

**Published:** 2025-01-17

**Authors:** Junbeom Kwon, Jungwoo Seo, Heehwan Wang, Taesup Moon, Shinjae Yoo, Jiook Cha

**Affiliations:** Seoul National University, Seoul, South Korea; Brookhaven National Lab, Upton, NY, United States

**Keywords:** resting-state fMRI, deep learning, task activation prediction, individual differences

## Abstract

Accurate prediction of the brain’s task reactivity from resting-state functional magnetic resonance imaging (fMRI) data remains a significant challenge in neuroscience. Traditional statistical approaches often fail to capture the complex, nonlinear spatiotemporal patterns of brain function. This study introduces SwiFUN (Swin fMRI UNet Transformer), a novel deep learning framework designed to predict 3D task activation maps directly from resting-state fMRI scans. SwiFUN leverages advanced techniques such as shifted window-based self-attention, which helps to understand complex patterns by focusing on varying parts of the data sequentially, and a contrastive learning strategy to better capture individual differences among subjects. When applied to predicting emotion-related task activation in adults (UK Biobank, n = 7,038) and children (ABCD, n = 4,944), SwiFUN consistently achieved higher overall prediction accuracy than existing methods across all contrasts; it demonstrated an improvement of up to 27% for the FACES-PLACES contrast in ABCD data. The resulting task activation maps revealed individual differences across cortical regions associated with sex, age, and depressive symptoms. This scalable, transformer-based approach potentially reduces the need for task-based fMRI in clinical settings, marking a promising direction for future neuroscience and clinical research that enhances our ability to understand and predict brain function.

## 1 Introduction

Task-based functional magnetic resonance imaging (fMRI) has been instrumental in cognitive neuroscience, offering insights into the functional neuroanatomy associated with adaptive and maladaptive cognition and behavior. This method holds promise for predicting individual cognitive functions and psychological disorders (Brodersen et al., 2011;Frässle et al., 2020), often outperforming resting-state fMRI in accuracy (Gal, Coldham, et al., 2022;Gal, Tik, et al., 2022;Greene et al., 2018;Sripada et al., 2020). Its utility extends to pre-surgical planning, where it aids in identifying functionally impaired brain regions, thus helping to reduce the risk of cognitive impairments post-surgery (Niu et al., 2021;Parker Jones et al., 2017). However, implementing task-based fMRI in practical settings such as clinical practice is challenging. This is due to issues such as ensuring participant compliance and motivation, as well as the necessity for strict experimental control. These issues become particularly pronounced in specific groups such as children, the elderly, and individuals with neurocognitive disorders or severe psychiatric conditions (Bernstein-Eliav & Tavor, 2024;Zhang et al., 2021).

As an alternative, recent research is steering toward predicting task reactivity using resting-state fMRI, which captures intrinsic brain activity patterns (Bernstein-Eliav & Tavor, 2024). This approach is based on the close relationships between resting-state and active-state functional networks (Cole et al., 2014;Elliott et al., 2019;Smith et al., 2009), suggesting that the “connectivity fingerprint” derived from resting-state fMRI reflects salient individual differences in cognitive functioning (Finn et al., 2015;Ito et al., 2022;Schultz et al., 2022). These fingerprints have been shown to predict task activation across various cognitive tasks (Cohen et al., 2020;Cole et al., 2016;Tavor et al., 2016;Tripathi & Somers, 2023;Zheng et al., 2022), even in patients with neurological disorders (Niu et al., 2021;Parker Jones et al., 2017;Tik et al., 2021). The consistent correlation between resting-state functional connectivity and task activation was validated across diverse sites, MRI vendors, and age groups in multiple cognitive tasks (Tik et al., 2023). In addition, task activation maps derived by resting-state functional connectivity achieved more accurate predictions of cognitive functioning than resting-state functional connectivity alone (Gal, Tik, et al., 2022). Furthermore, researchers can investigate underlying functional connections of task-related cognitive processes by identifying resting-state functional connectivity predictive of task-induced brain activity (Izakson et al., 2023;Tik et al., 2021).

The advent of deep learning marks a significant evolution in connectivity fingerprinting from fMRI data, enabling enhanced predictive accuracy and reliability. BrainSurfCNN, a surface-based deep convolutional neural network pre-trained on extensive datasets and fine-tuned for specific applications, has shown marked capability in predicting task activation maps from resting-state grayordinate fMRI data (Ngo et al., 2022;M. Nguyen et al., 2024). These models surpass traditional approaches that rely on rule-based features of the functional patterns (e.g., independent component analysis followed by dual regression (Tavor et al., 2016), or stochastic probabilistic functional modes (Zheng et al., 2022)), which only partially capture the brain’s spatial and temporal dynamics. Other recent studies suggested that applying deep learning models directly to volumetric fMRI data can better capture subtle individual differences in fMRI data than deep learning models relying on handcrafted features, showing outstanding predictive performance for cognitive and biological variables (Kim et al., 2023;Malkiel et al., 2022;S. Nguyen et al., 2020;Rosenman et al., 2024). The question then arises: Can we predict task-related activations from resting-state brain activity by capturing spatiotemporal patterns directly from fMRI data?

In response to this challenge, we introduce SwiFUN (Swin fMRI Transformer with UNET), a pioneering end-to-end deep learning model designed to generate task activation maps from resting-state fMRI data. SwiFUN, leveraging the innovative Swin (shifted window) UNETR architecture (Hatamizadeh et al., 2022) and a Swin 4D fMRI Transformer (Kim et al., 2023) combined with contrastive learning strategy (Ngo et al., 2022), represents the first application of such technology in human neuroimaging studies. Our findings suggest that SwiFUN can learn rich spatiotemporal representations from resting-state fMRI data, significantly improving the prediction of human brain activity during specific tasks. This novel approach may potentially transform neuroscience research, offering a more effective and inclusive method of exploring the functional neuroanatomy of cognition and behavior.

## 2 Methods

### 2.1 Experimental setup

#### 2.1.1 Data

We used 3-Tesla resting-state fMRI (rsfMRI) and task-based fMRI (tfMRI) data from participants in the UK Biobank (Sudlow et al., 2015) and the Adolescent Brain Cognitive Development study (Casey et al., 2018). In contrast to the conventional approach using rsfMRI data and tfMRI in surface space (CIFTI: (“grayordinate” surface + volume)) (Van Essen et al., 2013), we used minimally preprocessed rsfMRI data and volumetric z-score task activation maps related to specific tasks or visual stimuli. We masked out the irrelevant brain and nonbrain voxels using two brain atlas images to restrict the analysis to the comparable brain regions as the ConnTask, a machine learning model depending on parcellation (Gal, Coldham, et al., 2022;Gal, Tik, et al., 2022;Tavor et al., 2016;Tik et al., 2021,^2023^). Specifically, we employed 100 cortical parcels defined bySchaefer et al. (2018), which are assigned to one of the seven brain networks and widely used in the previous research (Cohen et al., 2020;Gal, Coldham, et al., 2022;Gal, Tik, et al., 2022;Tavor et al., 2016), and Harvard–Oxford cortical and subcortical structural atlases for evaluating the model’s performance in predicting subcortical brain activity (Desikan et al., 2006;Frazier et al., 2005;Goldstein et al., 2007;Makris et al., 2006). As a result, a total of 132,032 (Schaefer) and 146,025 (Harvard–Oxford) valid voxels in task activation maps and each rsfMRI volume were selected for our analysis.

##### 2.1.1.1 UK Biobank data

UK Biobank (UKB) is a large biomedical database that contains health-related information from half a million UK participants. To evaluate the model’s ability to generate task activation maps, we ran the analysis on the preprocessed rsfMRI and tfMRI of 7,038 individuals (age=54.971±7.53years, 52.7% female) from UK Biobank release 2. The detailed acquisition protocol and preprocessing process are described inMiller et al. (2016)andAlfaro-Almagro et al. (2018). The fMRI data have a resolution of 2.4 × 2.4 × 2.4 mm, having TR of 0.735 s and TE of 39 ms. The scan duration is 6 min (490 time points) for rsfMRI and 4 min (332 time points) for tfMRI. The initial preprocessing performed by the UKB Brain imaging team includes motion correction, group-mean intensity normalization, high-pass temporal filtering, and EPI warping. Spatial smoothing with a Gaussian kernel of FWHM 5 mm was applied to tfMRI scans before intensity normalization. Unlike tfMRI scans, the resulting rsfMRI scans were further ICA+FIX cleaned to remove structured artifacts (Beckmann & Smith, 2004;Salimi-Khorshidi et al., 2014). The task used is the Hariri faces/shapes “emotion” task, where participants viewed faces or shapes sequentially in each block of trials. Z-statistics of three contrasts were estimated from tfMRI using FSL FEAT (Woolrich et al., 2004): SHAPES-BASELINE, FACES-BASELINE, and FACES-SHAPES (Barch et al., 2013). The rsfMRI scans and task contrast maps were then registered to standard MNI space (Grabner et al., 2006).

##### 2.1.1.2 Adolescent Brain Cognitive Development data

Adolescent Brain Cognitive Development (ABCD) study is the largest longitudinal study of brain and cognitive development in the United States. We used tfMRI scans during emotional n-back (EN-back) task from 4,944 adolescents (age =9.95±0.63years, 49.7% female) (Casey et al., 2018) from release 2. The fMRI data have a resolution of 2.4 × 2.4 × 2.4 mm, having TR of 0.8 s and TE of 30 ms. The scan duration is 5 min (383 time points) for rsfMRI and 4.8 min (362 time points) for tfMRI. Using fMRIprep (Esteban et al., 2019), an automated preprocessing pipeline for structural and functional MRI, we conducted brain extraction, slice time correction, and confounds estimation. Then, we spatially normalized fMRI data to the standard MNI space for the pediatric brains (i.e., MNIPediatricAsym space;V. Fonov et al., 2011;V. S. Fonov et al., 2009). For rsfMRI, we additionally applied low-pass filtering, head movement correction, and artifact removal, regressing out signals from nongray matters (aCompcor). We applied spatial smoothing with a Gaussian kernel with FWHM of 5 mm to tfMRI and normalized the signal of each voxel by the mean across the time of each voxel followingChaarani et al. (2021).

We used a generalized linear model (GLM) to estimate the participants’ task-related brain activation map in the Emotional N-back task. We created a task model considering a block design followingChaarani et al. (2021), with a task block duration of 24.5 s and a fixation block duration of 14 s. We used a total of nine conditions of blocks as GLM regressors (i.e., block of 0-back positive face, 0-back negative face, 0-back neutral face, 0-back place, 2-back positive face, 2-back negative face, 2-back neutral face, 2-back place, and fixation). We used a total of 15 nuisance regressors for GLM, including confounding variables calculated by fMRIPrep, such as average signal within brain mask, white matter mask, and CSF mask, as well as six motion parameters and their time derivatives. We used SPM’s canonical hemodynamic response function (double-gamma SPM model with time derivative). We used high-pass filtering with a Discrete Cosine Transform (DCT) basis (cutoff freq = 1/128 Hz) to remove low-frequency fluctuations in the BOLD time series as the drift model. We censored frames with framewise displacement (FD) of 0.9 mm or higher. We estimated four z-statistics maps for the following contrasts: PLACES-BASELINE, FACES-BASELINE, FACES-PLACES, and 2BACK-0BACK. For brevity, comparisons between specific stimuli and the baseline condition are expressed using only the stimulus name (e.g., FACES-BASELINE is denoted as FACES).

##### 2.1.1.3 Feature extraction for resting-state functional modes

Previous studies have used resting-state functional modes for predicting task activation maps (Gal, Coldham, et al., 2022;Gal, Tik, et al., 2022;Tavor et al., 2016;Tik et al., 2021,^2023^;Zheng et al., 2022). Functional modes refer to consistent spatial patterns of brain activity observed across different individuals. Group-Independent Component Analysis (ICA) is widely used to extract the group-level functional modes from rsfMRI. The spatial maps from group-level ICA are data-driven parcellation that extracts independent components (IC) from fMRI data, each representing distinct brain functional networks. We used two versions of group-level ICs (25 and 100) provided by UK Biobank (refer toMiller et al. (2016)for the detailed process). The group-level parcellation results in UK Biobank are available at the following URL:http://biobank.ctsu.ox.ac.uk/crystal/refer.cgi?id=9028. We filtered out components considered artifactual from the initial 25 and 100 group-level ICs, leaving 21 and 55 ICs for the dual regression (Miller et al., 2016).

To obtain the group-level functional modes of ABCD data, we carried out group-ICA on the rsfMRI of ABCD data. Group ICA was performed only on the specific training subjects, and we excluded them from the rest-to-task fMRI activation map prediction. Only genetically unrelated European subjects with at least 300 rsfMRI time points among healthy controls were selected. Healthy controls were defined as subjects with a Child Behavior Checklist (CBCL) total score of 60 or less and a normal KSAD diagnosis based on parent and child questionnaires. This totaled 215 subjects, including 103 males and 112 females. The rsfMRI data were spatially smoothed with a 5 mm kernel. Then, the group-PCA was applied to the fMRI data by MELODIC’s Incremental Group-PCA, generating 1,000 spatial eigenmaps (Smith et al., 2014). The eigenmaps were used to generate group-ICA spatial maps at multiple ICA dimensions by using FSL’s MELODIC function. The ICA dimensions used for our experiments were 25 and 45.

To derive functional modes for each subject, we performed dual regression using the spatial group IC maps as templates (Nickerson et al., 2017). Before the dual regression, we masked the group IC maps and fMRI scans with a whole-brain mask to exclude unnecessary nonbrain voxels. The dual regression consists of two steps. In the first step, the fMRI data were regressed onto the spatial IC maps, resulting in the subject-specific time courses associated with each IC. In the second step, the previous subject-specific time courses were regressed onto the previous fMRI data, creating individual network-specific spatial IC maps. The individual network-specific spatial IC maps were then used for weighted seed-to-voxel analysis. The individual IC maps were used to regress against the individual rsfMRI time series, resulting in a single time series for each spatial map. Subsequently, each time series was correlated with the original fMRI data to generate connectivity maps for each spatial IC. The resulting connectivity features have voxels with different dimensions based on the number of ICs.

#### 2.1.2 Measures of model performance

We evaluated the predictive performance of task activation maps by assessing, firstly, the overall prediction accuracy and, secondly, how well an individual predicted map identifies individual variability as outlined in previous studies (Tavor et al., 2016;Tik et al., 2021;Zheng et al., 2022). The Pearson correlation coefficient served as our metric for comparing predicted and actual task activation maps.

Our analysis involved calculating pairwise correlation coefficients across N individuals’ actual and predicted activation maps, resulting in an N x N correlation matrix. The diagonal elements, denoted as$C_{ii}$, reveal the correlation between predicted and actual activation maps from the same individual (diagonal correlations). Conversely, the off-diagonal elements,$C_{ij}$(wherei≠j), represent the correlations between theith actual andjth predicted activation maps across different individuals (off-diagonal correlations). We evaluated overall prediction accuracy using the median of diagonal correlations, providing an overview of how well the predicted task activation maps correlated with the actual subject’s maps.

Assessing subtle differences in individual task activation maps can be challenging, as overall prediction accuracy might be inflated by merely predicting the group mean activation map for individuals performing the same task. Therefore, we used four metrics to compare how well each model captures individual differences in task activation maps: the diagonality index, the identification success rate, the diagonal percentile mean, and the effect size (D) of the Kolmogorov–Smirnov test. The diagonality index measures how well a model distinguishes between individual task activation maps. It is calculated by subtracting the mean of off-diagonal correlations (cross-subject correlations) from the mean of diagonal correlations (same-subject correlations). A higher diagonality index indicates the model captures individual differences effectively, while a lower index suggests the model may be overfitting to group-level patterns. This metric has been commonly used to assess individual-level accuracy in previous studies (Tik et al., 2021,^2023^;Zheng et al., 2022). Additionally, we conducted a Kolmogorov–Smirnov (KS) test on off-diagonal correlations and diagonal correlations to determine whether there was a significant difference in distributions between the cumulative density functions (CDF) of diagonal and off-diagonal correlations as done byTavor et al. (2016). The KS test is a nonparametric test better suited for comparing distributions that may not be normally distributed. We used the effect size of the Kolmogorov–Smirnov test (D), which represents the maximum difference between the CDFs of the diagonal and off-diagonal correlations, to compare the model’s performances.

We then tested the specificity of predicted maps with an identification success rate (Finn et al., 2015;Yoo et al., 2022). This metric evaluates whether the task activation map predicted by the model for a particular subject specifically predicts that subject’s actual activation compared with task activation maps predicted for other subjects. The formula for the identification success rate is as follows:

$$\text{Identification success rate}=\frac{\text{Number of correctly identified subjects}}{\text{Total number of subjects}}.$$

The identification success rate is a conservative metric as it only determines cases where the similarity between the predicted map and the actual map is the highest among all predicted maps, making it difficult to judge overall specificity. To address this limitation, we developed a new metric termed the diagonal percentile mean. The diagonal percentile mean is determined by calculating the average percentile of each subject’s diagonal correlation compared with their off-diagonal correlations. The formula for the diagonal percentile mean is as follows:

$$\text{Diagonal percentile mean}=\frac{1}{N}\sum_{i=1}^{N}\left(\frac{\text{Number of}\;C_{ij}\text{ less than}\;C_{ii}}{\text{Total number of }C_{ij}\text{ for subject }i}\right),$$

where$C_{ij}$is the correlations between theith actual andjth predicted activation maps across N different individuals. This metric ranges from 0.5 (indicative of by chance) to 1. If the predicted map for a subject shows lower agreement with the actual map compared with the predicted maps of the others, the diagonal percentile would be closer to 0.5. If there is a high degree of agreement compared with the predicted maps of the others, the diagonal percentile for the subject would be closer to 1. These metrics may provide a comprehensive evaluation of the predicted activation maps in terms of overall prediction accuracy and individual-level specificity.

### 2.2 Swin fMRI UNet Transformer (SwiFUN)

We examined how much information can be extracted from volumetric fMRI data, which had not been previously analyzed within this field of study. Unlike surface-based fMRI data, which projects three-dimensional volumetric data onto a two-dimensional cortical surface, volumetric fMRI data preserve the three-dimensional spatial relationships, potentially facilitating the model’s ability to learn spatial adjacency. To explore this, we developed a novel deep learning framework called Swin fMRI UNet Transformer (SwiFUN), which can generate task activation maps from spatiotemporal representations in rsfMRI data. SwiFUN is based on the architecture of the Swin UNet TRansformer (UNETR) model proposed for brain structural segmentation (Hatamizadeh et al., 2022). We adopted the Swin UNETR module from MONAI (Cardoso et al., 2022). As shown inFigure 1, for each contrast, a separate SwiFUN model was trained using a series of fMRI volumes (Ttime points) as input. The goal of each SwiFUN model was to learn the spatiotemporal patterns from the rsfMRI data for predicting a single 3D task activation map. The intermediate outputs of each Swin Transformer layer are fed into the UNET decoder through skip connections. This UNET structure enhances training stability and facilitates the generation of higher resolution image information.

**Fig. 1.**
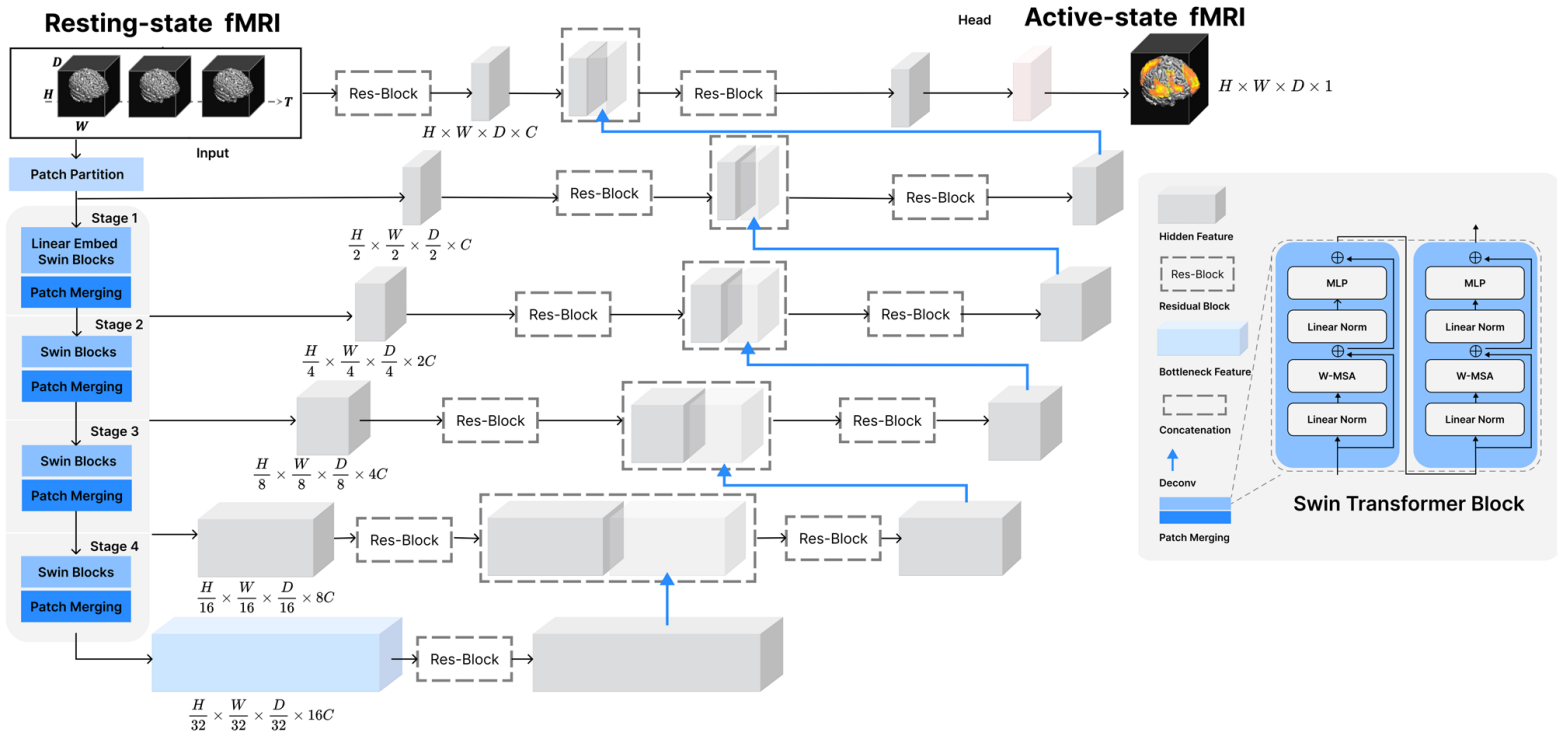
The overall architecture of Swin fMRI UNet Transformer (SwiFUN). SwiFUN takesTtime points of resting-state fMRI volumes as input and predicts a three-dimensional task activation map. Time dimension (T) is considered channel dimension at the first stage. The figure is adapted from Swin UNETR (Hatamizadeh et al., 2022).

SwiFUN was trained using datasets divided into training, validation, and testing sets with a distribution ratio of 70% for training, 15% for validation, and 15% for testing. We iteratively trained the model with three different splits. We used the test set for visualization, variable prediction, and correlation analysis with head motion. To train SwiFUN, we used a dropout rate of 0.3 and an embedding size of 24 (denotesCinFig. 1). Our model was trained using the AdamW optimizer with a learning rate 5e-5, combined with a Cosine Annealing Warmup Restart scheduler, for 10 epochs. We optimized our model using mean squared error (MSE) loss. Additionally, we employed the Reconstruction-Contrastive loss to improve the model’s performance in identifying individual differences (Ngo et al., 2022). Due to memory constraints in GPU (Nvidia A100 40GB), using an entire run of the resting state fMRI volumes (e.g., 490 volumes in UKB) as a single input was not feasible. Instead, we partitioned the fMRI volumes into subsequences of 30 volumes (time points) each. During training, the model learned to predict a task activation map based on these subsequences. When predicting a subject’s activation map for evaluation, the model first computed task activation maps for each subsequence, which were then averaged to produce the final task activation map for that subject. We used a mini-batch size of 4 (each containing subsequences) during the experiments. We also assessed the impact of the input subsequence length on prediction performance, as shown inSupplementary Figure S1, which depicts the effect of sequence length and mini-batch size on the performances.

### 2.3 Reconstruction-Contrastive loss

Prior studies used a contrastive loss to make the predicted activation map of a subject closely resemble their actual map while ensuring it significantly differs from the maps of other subjects (Ngo et al., 2022). We tailored this method to balance two key goals: achieving overall similarity (overall prediction accuracy) and ensuring distinct subject recognition (individual identification). InSupplementary Figure S2, we observed a trade-off between the overall prediction accuracy (measured by diagonal median) and individual identification (measured by diagonality index) during training, which supports the use of the RC (Reconstruction-Contrastive) loss.

Our loss function introduces two novel designs. Firstly, unlike previous studies that calculated the Reconstruction-Contrastive Loss$L_{RC}$using just two subjects at a time (Ngo et al., 2022), we expanded this comparison to include four or more subjects. We ascertained that multiple subsequences of the same subject within a batch were not included in$L_{C}$, ensuring that$L_{C}$focuses on differences between subjects. Secondly, whereas previous methods used a two-step process of training until the same-subject error$L_{R}$converges and then applying$L_{C}$at certain points afterward, we introduced a new parameterλ, which only considers the relative weight of the two and allows for end-to-end training.

$$L_{R}=\frac{1}{N}\sum_{i=0}^{n}d\left({\hat{x}}_{i},x_{i}\right),L_{C}=\frac{1}{N^{2}-N}\sum_{x_{j}\in B_{i},j\neq i}d\left({\hat{x}}_{j},x_{i}\right)$$

$$L_{RC}=\lambda L_{R}-(1-\lambda )L_{C}.$$

The Reconstructive-Contrastive loss$L_{RC}$is defined as follows: Given a mini-batch of N samples B, where each sample$x_{i}$represents the target 3D task activation image of a subjecti, and$\hat{x}$represents the corresponding prediction.$N^{2}-N$in$L_{C}$loss denotes the number of all possible pairs between predicted maps and actual maps from different samples in a batch.d()is the distance function, the mean square error (MSE) in this experiment.

### 2.4 The baseline model

#### 2.4.1 Conntask

Previous studies have used a GLM-based model, ConnTask, to predict task activation maps from resting-state functional modes (Gal, Tik, et al., 2022;Tavor et al., 2016;Tik et al., 2021). These studies used grayordinate fMRI data (in CIFTI); however, in our study, we used the volume data (masked and converted into vectors) for a fair comparison with SwiFUN. We trained 100 generalized linear models, each corresponding to 1 of the 100 cortical parcels defined in Schaefer’s atlas (Schaefer et al., 2018). These models predicted task activation maps from connectivity features (Independent Components) associated with each parcel. In the task activation maps, each region of interest (ROI) was predicted from the connectivity features of its corresponding ROI. We only used independent components as input features, treating the voxels in the connectivity features as independent training samples.

After training the models, represented by$\beta_{k}$, we averaged$\beta_{k}$across all subjects during the inference phase. We employed a 5-fold cross-validation approach, iteratively training the models with 80% of the subjects and using the remaining 20% to predict their task activation maps. ConnTask’s fivefold cross-validation was performed within SwiFUN’s test set. Our study also explored how the number of independent components affects the predictive performance (Supplementary Table S1).

#### 2.4.2 BrainVolCNN

Ngo et al. (2022)predicted task activation maps using BrainSurfCNN, a model designed for surface-based fMRI. However, a direct comparison between BrainSurfCNN and our volumetric SwiFUN model presents challenges, given the surface-mesh framework of BrainSurfCNN. For a fair comparison, we designed Brain “Vol” CNN, a volumetric adaptation of BrainSurfCNN. In this adaptation, we replaced the original mesh-based components with standard 3D convolutions and pooling operations, while retaining the U-Net architecture with skip connections for multiscale feature processing. This modification allows BrainVolCNN to effectively handle volumetric fMRI data. For consistency, we trained BrainVolCNN using the same settings as SwiFUN, with mean squared error (MSE) as the loss function.

#### 2.4.3 Test–retest contrasts

The UK Biobank (UKB) dataset includes data from repeat visits. We assessed the correlation between the task activation maps of releases 2 and 3 for the 577 participants who were present in both releases, selected from a larger pool of 7,038 datasets initially used in release 2. The correlation between the task activation maps from these two releases serves as a measure of the test–retest reliability of the actual contrast map, setting an upper bound of the model performance. The ABCD dataset includes two fMRI runs for the same emotional n-back task, both scanned on the same day. We computed a metric for test–retest reliability, similar to the UKB dataset, for the 4,934 participants who had activation maps from both runs.

### 2.5 Relationship between head motion and prediction accuracy of task activation maps

We experimented to estimate the factors contributing to the predicted maps’ overall quality. We evaluated whether the overall head motion levels of fMRI scans are correlated with the prediction accuracy of predicted maps. In the ABCD dataset, we used averaged frame-wise displacement (FD) to measure the overall head motion of resting-state and task-based fMRI scans. In the UKB dataset, we used the mean head motion of resting-state (field 25741) and task-based fMRI scans (field 25742) in millimeters (mm) averaged across space and time points.

### 2.6 Prediction of individual traits from task activation maps

We evaluated the predicted task activation maps by assessing how well the predicted task activation maps predicted individual traits. First, the predicted task activation maps were flattened after removing nonbrain voxels using Schaefer’s atlas (Schaefer et al., 2018). The test set of SwiFUN was divided into an 80% train set and a 20% test set, and the performance was averaged over 20 iterations. To extract important features from the 132,032 valid voxels, feature reduction was performed using PCA, and the number of principal components was determined as the number with 90% explained variance. For classification and regression tasks, logistic regression with l2 regularization was used. UKB FACES, SHAPES, and FACES-SHAPES contrast were analyzed. Sex was used as the target variable for the classification task, while age, mild and severe depression (given by PHQ-9 score (Manea et al., 2012)), and neuroticism (given by N-12 score, field 20127) were used as target variables for the regression task. The performance of the classification task was measured by the AUROC and accuracy between predicted and actual values. The performance of the regression task was measured by Pearson correlation (r), prediction$R^{2}$(Scheinost et al., 2019;Yoo et al., 2022), and mean square error (MSE). We conducted a permutation test to determine whether the differences between the individual trait prediction performance of ConnTask, BrainVolCNN, SwiFUN with MSE and RC loss, and the actual task activation map were significant. As performed inGal, Coldham, et al. (2022), we randomly shuffled the prediction accuracies of each model and calculated the group differences between them. The*p*-value was determined as the number of cases where the group difference determined by chance was higher or equal to the actual difference between the performances of two models, divided by the total number of permutations (10,000). The*p*-value was then Bonferroni corrected for 10 multiple comparisons among 5 types of activation maps (real, ConnTask, BrainVolCNN, and SwiFUN with MSE loss, and RC loss).

## 3 Results

### 3.1 Performances comparison in predicting task activation maps

**Fig. 2.**
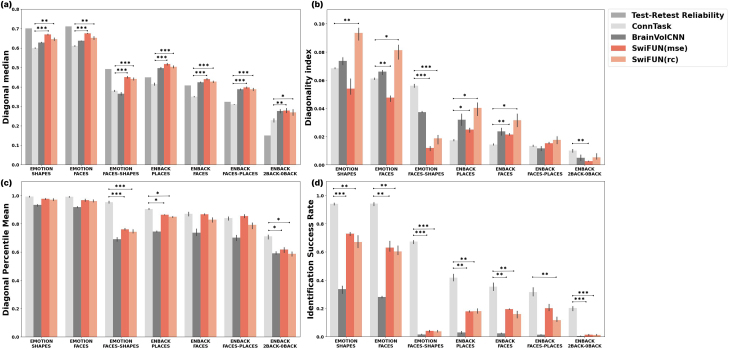
Performance comparisons of SwiFUN and baseline models in UK Biobank and ABCD contrasts. Deep learning models (BrainVolCNN and SwiFUN) used volumetric rsfMRI data as input, while ConnTask employed connectivity features. (a) The diagonal median represents the overall prediction accuracy of each model. (b) The diagonality index, (c) diagonal percentile mean, and (d) identification success rate represents the model’s ability to capture individual differences. SwiFUN (mse) refers to SwiFUN trained with mean square error (MSE) loss, whereas SwiFUN (rc) indicates SwiFUN trained with Reconstruction-Contrastive (RC) loss. Bar heights represent the mean values across three repetitions, and the error bars show a 95% confidence interval. Bonferroni-corrected*p*-values for comparisons between ConnTask and SwiFUN models are described as asterisks over bar plots (*p< 0.05, **p< 0.01, and ***p< 0.001).

We evaluated the efficacy of SwiFUN in predicting task activation maps from the UK Biobank (UKB) and Adolescent Brain Cognitive Development (ABCD) datasets, comparing its performance with ConnTask, BrainVolCNN, and test–retest reliability (Fig. 2). Our analysis included a comparison between two loss functions utilized by SwiFUN: mean absolute error (MSE) and Reconstructive-Contrastive (RC) loss. The weight of the contrastive loss term (1−λ) in RC loss was specified as 0.66 (refer toSupplementary Figure S3to find the effect of the contrastive loss term). To compare the performance of ConnTask, BrainVolCNN, and the two SwiFUN models, we conducted two-sided t-tests to evaluate statistically significant differences. To account for multiple comparisons,*p*-values were Bonferroni corrected for the six pairwise comparisons among the four models. Across all contrasts within both datasets, SwiFUN models showed significantly higher diagonal median performance than ConnTask (Fig. 2a). SwiFUN trained with MSE loss showed a significantly higher diagonal median than BrainVolCNN in UKB contrasts (p<0.001) and a similar diagonal median in ABCD contrasts. In UKB contrasts, SwiFUN’s overall accuracy was comparable with the test–retest reliability, and its accuracy surpassed the test–retest reliability of the ABCD contrasts. SwiFUN trained with MSE loss exhibited higher diagonal median performance than its RC loss counterpart in all contrasts.

We then assessed the models’ ability to capture individual differences in task activation maps using a diagonality index (Fig. 2b). SwiFUN generally exhibited a higher diagonality index when trained with RC loss than with MSE loss. SwiFUN trained using RC loss showed a higher diagonality index than ConnTask in the UKB SHAPES (p<0.01) and FACES (p<0.05) contrasts. In ABCD PLACES and FACES contrasts, both types of SwiFUNs observed a higher diagonality index than ConnTask (p<0.05). However, in the UKB FACES-SHAPES contrast, SwiFUN models showed a significantly lower diagonality index than ConnTask (p<0.001). Furthermore, we conducted a Kolmogorov–Smirnov test to assess the statistical significance of the disparity between the cumulative distribution functions (CDF) of diagonal and off-diagonal correlations. InSupplementary Figure S4a, regardless of the loss type, SwiFUN exhibited a significant distinction between diagonal and off-diagonal correlations in all task contrast maps (p<0.001), except a 2BACK-0BACK contrast from ABCD data. For all contrast maps, using the RC loss led to larger effect sizes (D) from the Kolmogorov–Smirnov test than the MSE loss. Overall, the magnitude of the effect size (D) was similar to that of the diagonality index (Fig. 2b).Supplementary Figure S4bshows why SwiFUN trained with RC loss has a higher effect size (D) in the Kormogorov–Smirnov test than the model trained with MSE loss. In UKB SHAPE contrast, the diagonal correlations of MSE loss were significantly lower than RC loss’s (D = 0.122,p<0.001). Still, the off-diagonal correlations of RC loss were much lower overall than MSE loss’s (D = 0.232,p<0.001). This means that RC loss maximizes the individual uniqueness of predicted task contrast maps by decreasing off-diagonal correlations to a greater extent than the decrease in diagonal correlations, resulting in an overall improvement in the specificity of the predicted maps.

InFigure 2c, SwiFUN models exhibited a significantly higher diagonal percentile mean than BrainVolCNN across all contrasts (p<0.05), except the ABCD 2BACK-0BACK contrast. SwiFUN’s diagonal percentile mean was comparable with ConnTask in most contrasts, except for those representing differences between conditions, such as the FACES-SHAPES contrast (UKB) and the 2BACK-0BACK contrast (ABCD). SwiFUN models demonstrated a significantly lower diagonal percentile mean than ConnTask in the UKB FACES-SHAPES contrast (p<0.001), as well as in the ABCD PLACES (p<0.05) and ABCD 2BACK-0BACK (p<0.05) contrasts.

Figure 2dpresents the identification success rate as a more conservative measure of specificity than the diagonal percentile mean. During the transition from diagonal percentile mean to identification success rate, ConnTask experienced an average performance reduction of 37.44%. In contrast, SwiFUN models showed a more pronounced decline of 67.1% (MSE) and 69.94% (RC), while BrainVolCNN exhibited the largest decrease, at 87.15%. Accordingly, SwiFUN models achieved significantly higher identification success rates than BrainVolCNN across all contrasts (p<0.01), except for UKB FACE-SHAPES and ABCD 2BACK-0BACK. However, ConnTask outperformed both SwiFUN models and BrainVolCNN, showing significantly higher identification success rates across all contrasts (p<0.01).

### 3.2 Qualitative evaluation on volumetric task activation map

**Fig. 3.**
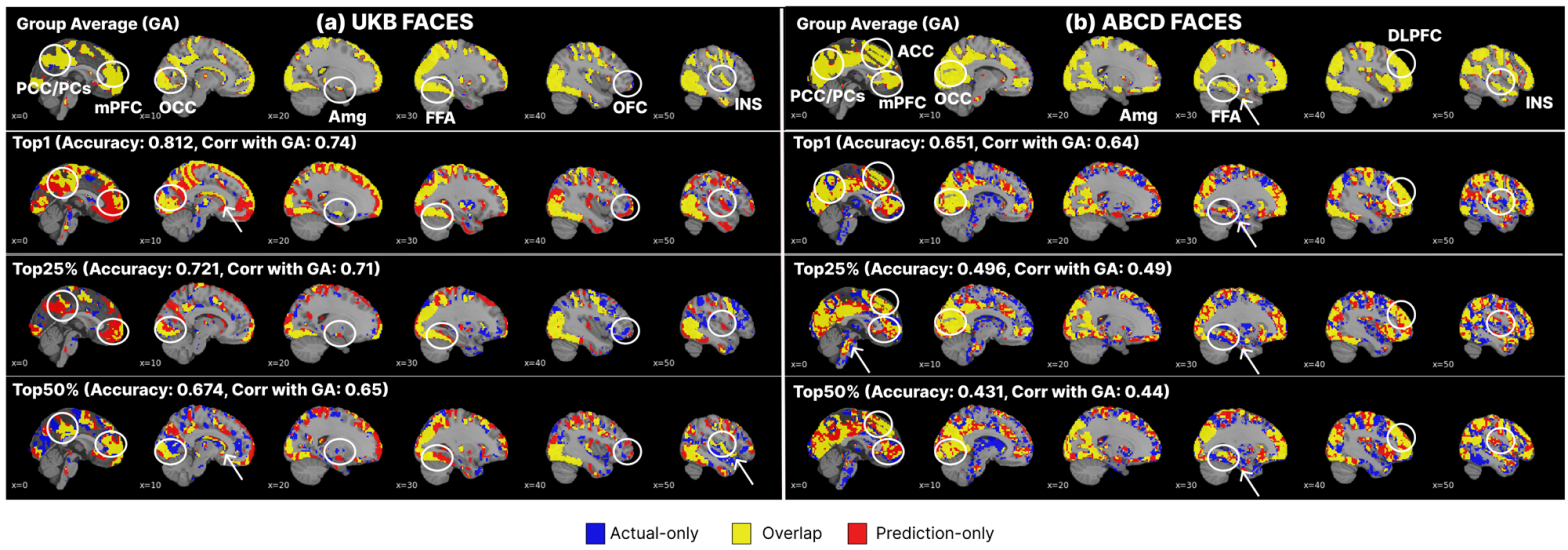
FACES contrast maps from (a) UKB and (b) ABCD tfMRI data in a series of sagittal views estimated by SwiFUN. Voxel values were normalized using z-scoring and then thresholded at the 97th percentile (two-sided). The yellow regions are cases where the areas predicted by SwiFUN match the actual activation, the blue regions are actual activations that SwiFUN did not predict, and the red regions are cases where SwiFUN predicted activation but no actual activation occurred. Accuracy indicates the correlation between the predicted and actual task activation map, and Correlation with GA means the correlation between the group-averaged map and the actual map. The brain voxels in the Harvard–Oxford atlas were used for visualization. White circles indicate brain regions prominently shown in the group-averaged maps, and white arrows represent the brain regions showing individual differences captured by SwiFUN. Abbreviations of brain regions are as follows: posterior cingulate cortex (PCC), medial prefrontal cortex (mPFC), amygdala (Amg), fusiform area (FFA), orbitofrontal cortex (OFC), insula (INS), dorsolateral prefrontal cortex (DLPFC).

Figure 3displays FACES contrast maps in the emotional matching task (UKB) and emotional n-back task (ABCD), showing group-averaged task activation map and individual maps of three subjects with the top-1, top 25%, and top 50% correlation between the actual and predicted maps using SwiFUN with MSE loss. For the FACES contrasts in both datasets, SwiFUN successfully predicted the activations observed in regions related to face recognition, such as the occipital lobe, fusiform face area, and amygdala. Additionally, as the white arrows show, SwiFUN effectively captured the individual differences within the brain regions.

The average of the actual task activation maps and the average of the task activation maps predicted by SwiFUN were highly consistent (r= 0.99 for both contrasts). However, while the actual activation maps showed low similarity to the representative group-averaged maps (average*r*= 0.625 for UKB FACES and*r*= 0.4 for ABCD FACES contrasts), the predicted activation maps exhibited patterns similar to the group-averaged maps (average*r*= 0.949 for UK Biobank data and*r*= 0.96 for ABCD data). Therefore, the more similar the actual activation maps were to the representative activations, the more accurately SwiFUN tended to predict those activation maps (*r*= 0.981 in UK Biobank data and*r*= 0.991 in ABCD data).

### 3.3 Head motion is negatively correlated with prediction accuracy of SwiFUN

**Fig. 4.**
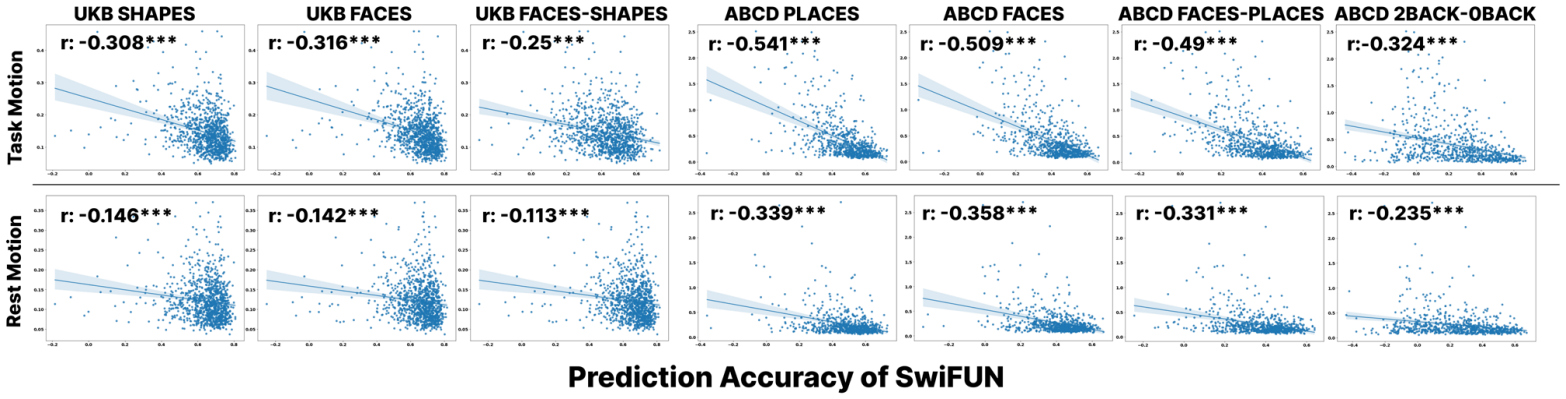
Scatter plots showing a negative correlation between mean head motion and prediction accuracy of SwiFUN. Each row represents the averaged head motion of task-based fMRI and resting-state fMRI. Prediction accuracy means the correlation between the predicted and actual task activation map. Asterisks indicate statistical significance of Pearson Correlation: *p< 0.05, **p< 0.01, and ***p< 0.001.

We verified that transient noise sources such as head motion in fMRI data are significantly associated with how well the task activation map reflects representative activation and the predictive model’s accuracy.Figure 4shows that SwiFUN’s prediction accuracy, as measured by the diagonal median, is significantly and negatively correlated with head motion level (p<0.001). In both the UK Biobank and ABCD datasets, the mean head motion level in task fMRIs (overall mean of -0.391) was more negatively correlated with the model’s performance than the mean head motion level in rsfMRIs (overall mean of -0.2377). We found the difference in correlation coefficient between task fMRI and rsfMRI data was significant in all contrasts through Fisher’s Z-Transformation (p<0.001). In addition, the Pearson correlation between head motion and prediction accuracy in the contrasts featuring overall brain activity (i.e., FACES, SHAPES, and PLACES) (mean of -0.3322) was stronger than the negative correlation (mean of -0.2905) in the region-specific contrasts (i.e., FACES-SHAPES, 2BACK-0BACK). The correlation between SwiFUN’s prediction accuracy and head motion showed an overall stronger negative correlation than the corresponding correlation in the ConnTask, but the difference between the two predictive models was not significant (Supplementary Figure S5). We also found that the higher each subject’s head motion, the lower the similarity between the actual task activation map and the group-averaged map (Supplementary Figure S6).

### 3.4 Prediction of individual traits from the predicted task activation maps

We evaluated how well task activation maps predicted by predictive models captured individual differences by assessing their accuracy in predicting sex, age, depression severity (measured by PHQ-9 scores), and neuroticism levels (measured by N-12 scores) from task activation maps derived from the UKB data. FromFigure 5a and b, the SwiFUN models outperformed both the actual task activation maps and the maps predicted by baseline models in identifying sex and age across all contrasts (p<0.001). For predicting depressive symptoms, as shown inFigure 5c, activation maps predicted by SwiFUN models showed better performance for the SHAPES (averager=0.153) and FACES (averager=0.119) contrasts than for the FACES-SHAPES contrast (r=− 0.012). The activation maps predicted by the SwiFUN models trained with RC loss exhibited a significantly higher Pearson correlation in predicting depressive symptoms than those predicted by ConnTask across all contrasts (p<0.001). Moreover, the activation maps from SwiFUN showed a significantly higher Pearson correlation with the actual maps for the SHAPES (p<0.05) and FACES-SHAPES (p<0.01) contrasts. For predicting neuroticism scores, SwiFUN models outperformed ConnTask across all contrasts (p<0.05). However, compared with actual task activation maps, SwiFUN showed significantly better predictive performance only for the FACES contrast (p<0.01). While ConnTask outperformed actual maps in predicting sex (p<0.001), it exhibited comparable or lower prediction performances than actual maps for age, depression, and neuroticism. The performance differences between SwiFUN models trained with mean squared error (MSE) loss and RC loss were insignificant across all variables and contrasts. Additional performance metrics for each prediction—accuracy for classification, and R² score and mean squared error for regression—are provided inSupplementary Table S2.

**Fig. 5.**
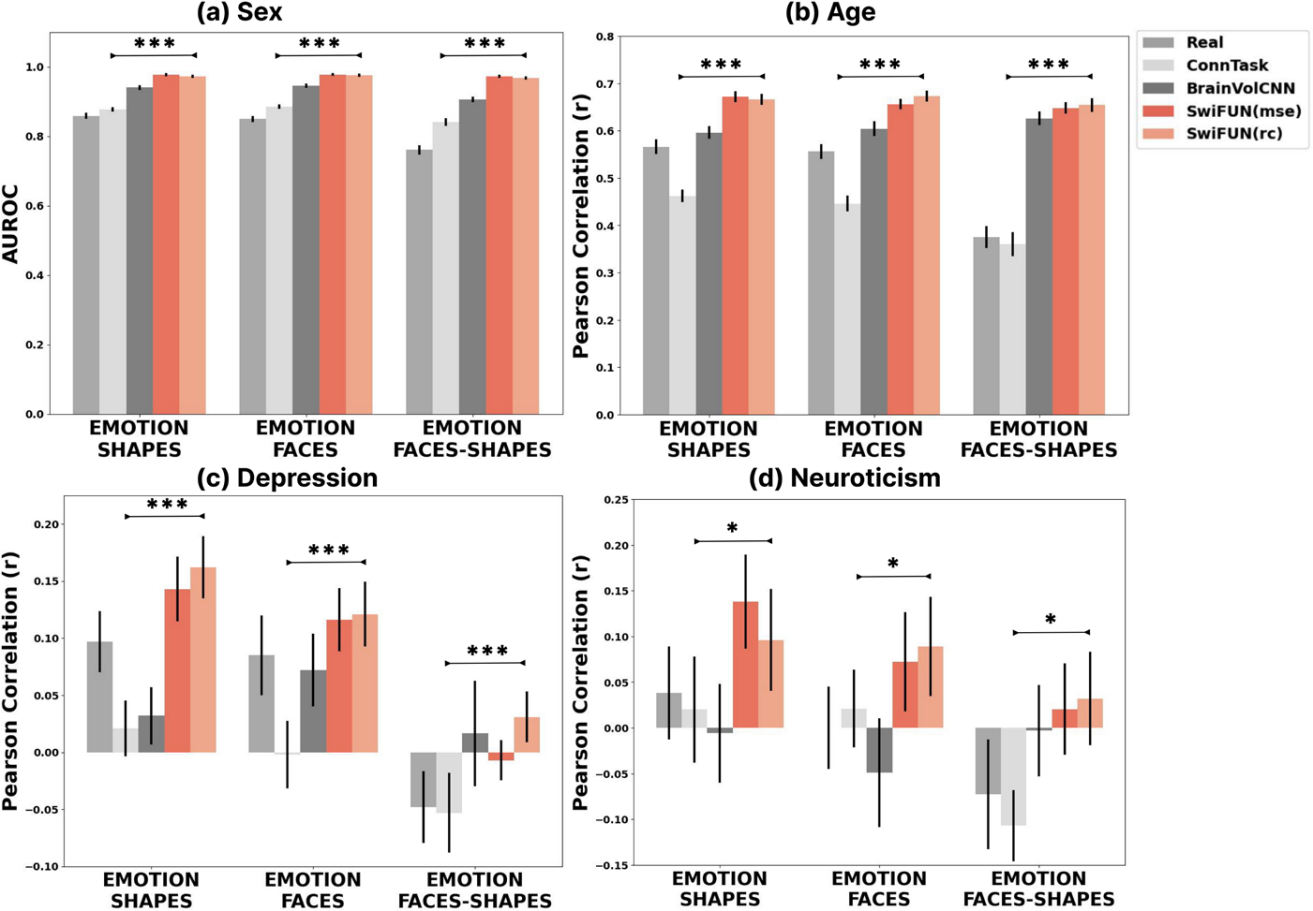
Predictive performance of actual and predicted task activation maps in UK Biobank data for individual traits; (a) sex, (b) age, (c) depression, and (d) neuroticism. The height of each bar represents the mean value in 20 repetitions, and the error bars show a 95% confidence interval. Bonferroni-corrected*p*-values for comparisons between ConnTask and SwiFUN (rc) are described as asterisks over bar plots (*p< 0.05, **p< 0.01, and ***p< 0.001).

## 4 Discussion

In this study, we introduce SwiFUN, a novel deep neural network that merges the fMRI Transformer’s capacity to learn spatiotemporal patterns from rsfMRI data with the predictive capabilities of the U-Net architecture. This combination enables the accurate prediction of task-specific brain activations. Unlike previous models that utilized surface fMRI data through complex processing pipelines, SwiFUN employs a more universally applicable end-to-end deep learning approach with volumetric fMRI data. By leveraging resting-state and task-related fMRI data from the ABCD and UKB datasets, SwiFUN outperforms the existing GLM-based model (ConnTask) and deep learning model (BrainVolCNN) in predicting task-related brain activity. Furthermore, the predicted task activation map can further predict an individual’s biological and psychological traits. Our approach offers new possibilities for studying brain (dys-)function without relying on laborious feature engineering processes.

SwiFUN exhibits the potential to predict functional activation patterns with high accuracy. In adults, the UKB data, the overall accuracy of SwiFUN was comparable with the test–retest reliability, indicating that the model’s performance was on par with the inherent stability of the functional data. In ABCD contrasts, SwiFUN demonstrated higher accuracy than the test–retest reliability. This unexpected finding may be related to the challenges in ABCD task fMRI data, for example, suboptimal experimental designs, head movement, and decreased attention in youth (Casey et al., 2018). These factors can lead to noisy and lower quality task activation maps. SwiFUN’s superior performance suggests that it may be particularly valuable for inferring functional activation patterns in datasets with suboptimal data quality, including those involving clinical populations or pediatric samples.

Consistent with the literature (Zheng et al., 2022), all the predictive models demonstrated lower individual identification performance for contrasts reflecting the difference between two conditions (e.g., FACES-SHAPES, FACES-PLACES, 2BACK-0BACK) compared with single-condition contrasts (e.g., FACES, SHAPES) (Fig. 2). This gap was more pronounced for the SwiFUN model than for the ConnTask model. One possible explanation might be the differences in how these two models leverage rsfMRI information to predict task-based activations. While ConnTask utilizes only the functional networks corresponding to each seed region (through ICA modeling) as the input, SwiFUN uses the spatiotemporal relationships among the entire brain regions (through shifted-window multi-head self-attention). For the contrasts that broadly capture the activity of the widespread brain regions, SwiFUN may have advantages; however, for the contrasts recruiting more focal brain activity (differences) (e.g., amygdalar activity differences in FACES-SHAPES), the additional information from the whole-brain resting-state data may not confer the same benefit.

We observed the characteristics of task activation maps that are less similar to the group-averaged map and their impact on SwiFUN’s prediction accuracy. InFigure 3, SwiFUN tended to predict the task activation maps less accurately when the task activation maps are unique, which are dissimilar to the group-averaged map. The more unique task activation maps were, the higher the head motion level their original resting-state or task-based fMRI had, as demonstrated inSupplementary Figure S6. Furthermore, we found that the level of head motion in the task fMRI data, which corresponds to the ground truth of the prediction, has a much more negative impact on the model’s prediction performance than the head motion in the input resting-state fMRI data. This relationship between head motion and the model’s predictive performance suggests that the reason for the model’s low prediction performance for certain subjects is transient factors such as the low quality of the actual activation maps.

Our results underscore SwiFUN’s capacity to elucidate individual characteristics, including depression, neuroticism, sex, and age. This is consistent with previous findings that task activation maps predicted by resting-state fMRI better predict cognitive variables such as intelligence than real task activation maps (Gal, Coldham, et al., 2022;Gal, Tik, et al., 2022). This advancement suggests that resting-state fMRI-derived task activation maps, particularly those generated by SwiFUN, hold significant potential to reflect cognitive and biological traits more accurately than traditional task activation maps, which may be under the influence of nuisance variables (head motion, scanning artifacts, attention level fluctuations) (Bernstein-Eliav & Tavor, 2024). Such capability implies broader applications for SwiFUN, including the potential for diagnosing and predicting psychiatric disorders, thereby positioning it as a valuable tool in neuroscientific research and clinical practice. Additionally, SwiFUN’s framework, leveraging an attention mechanism-based deep neural network, is well equipped to integrate additional imaging modalities, such as T1-weighted structural MRI or diffusion-weighted MRI. This capability may not only enrich its application in neuroimaging analysis but also specifically bolster its potential for more comprehensive and nuanced modeling of brain function and dysfunction.

Naturalistic fMRI, such as using movies as stimuli, offers a powerful lens for studying brain function in real-world contexts, potentially outperforming traditional methods (resting-state fMRI or task-based fMRI) in predicting individual traits and mapping brain activity (Bernstein-Eliav & Tavor, 2024;Finn & Bandettini, 2021;Gal, Coldham, et al., 2022). However, the dynamic nature of naturalistic stimuli poses challenges for conventional fMRI analysis techniques. SwiFUN, with its ability to capture spatiotemporal changes in brain activity, may emerge as a promising solution for unlocking the rich information embedded within naturalistic fMRI data.

Our study faces three primary limitations. First, understanding which brain regions significantly affect the prediction of task activation maps is a challenge. Previous research has linked task-related brain activity with specific resting-state functional connectivity using generalized linear models (Izakson et al., 2023;Tik et al., 2021). However, SwiFUN’s use of multiple nonlinear transformations complicates the clarity of these input–output relationships. In fMRI studies utilizing deep neural networks, Explainable AI (XAI) methods such as Grad-CAM and Integrated Gradients are widely used to pinpoint brain areas relevant to the single target outcome (Kim et al., 2023;S. Nguyen et al., 2020). However, these techniques are not yet adept at dissecting spatiotemporal features of four-dimensional resting-state fMRI inputs attributing to the resultant three-dimensional task-related activation maps. Thus, creating effective interpretation methods for SwiFUN remains a crucial future objective. Second, due to hardware limitations, the current model cannot process the entire fMRI sequence at once, with our Nvidia A100 40GB GPU managing only up to 30 volumes simultaneously. This limitation hinders the model’s ability to capture extended brain dynamics, which can last for significant periods. Enhancing the model to accommodate longer input sequences represents a vital future goal. Lastly, a separate model must be trained for every distinct task and condition, which is not only resource intensive but also overlooks potential commonalities across various tasks and conditions (Ngo et al., 2022).M. Nguyen et al. (2024)have recently shown that providing resting-state functional connectivity and group-activation maps as inputs to deep learning models can enable the prediction of individualized activation maps. Likewise, developing learning strategies that can be applied to diverse tasks and conditions through a single training process is a crucial challenge for future research.

## 5 Conclusion

This study suggests that training deep neural networks that capture spatiotemporal patterns directly from fMRI data may contribute to better performance in task activation prediction. In the future, we anticipate predicting various task-related brain activity from just a few minutes of resting-state fMRI data, significantly reducing the scanning time and effort required to capture task-based fMRI.

## Supplementary Material

Supplementary Material

## Data Availability

The access procedures of the Adolescent Brain Cognitive Development (ABCD) study and UK Biobank specify that only approved researchers can access participant data. Therefore, the data from this study cannot be publicly released. The code for SwiFUN and BrainVolCNN is available athttps://github.com/Transconnectome/SwiFUN. The code to run our baseline ConnTask is available athttps://github.com/ShacharGal/connTask.
