# Supplementary material for: Predicting task-related brain activity from resting-state brain dynamics with fMRI Transformer
Source: Imaging Neurosci (Camb). 2025 Jan 17;3:imag_a_00440. doi: 10.1162/imag_a_00440 (PMC12320000; doi:10.1162/imag_a_00440)
Supplement: Supplementary Material [file imag_a_00440-supp.pdf]

Supplementary Material

1. Effect of input sequence length and batch size on the predictive performance of SwiFUN

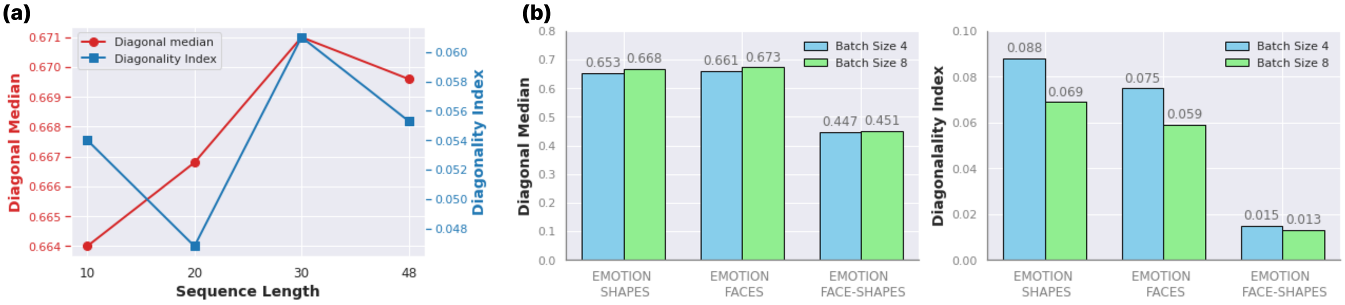

Figure S1: Effect of sequence length and batch size on the performance of SwiFUN

In Figure S1 (a), we confirmed whether the sequence length impacts the overall concordance (diagonal median) and individual identification (diagonality) of SwiFUN. SwiFUN was trained on the SHAPES contrast map using MSE loss. The experiments were conducted with a fixed mini-batch size of 4, and four different settings were tested with lengths of 10, 20, 30, and 48 considering the limitations of GPU memory (Nvidia A100 40GB GPU). The overall performance differences were not substantial, but the diagonal median showed improvement as the sequence length increased, reaching its peak at length 30. On the other hand, the diagonality index displayed the best performance at length 30 but did not exhibit consistent improvement with increasing length. Considering these results and the training speed associated with sequence length, all experiments were conducted with a length of 30.

Furthermore, we confirmed whether SwiFUN trained with RC loss is impacted by the mini-batch size (Figure S1 (b)). Considering that the contrastive loss compares samples within the mini-batch, we hypothesized that the mini-batch size would impact performance. In the RC loss, we set the value of $1-\lambda$ (the weight of the contrastive loss term) to 0.33. Our analysis revealed that in all tasks, the diagonal median exhibited a small increase as the batch size increased. On the other hand, the diagonality index showed a decrease with larger batch sizes. This suggests that increasing the number of samples in a batch can weaken the effect of contrastive loss.

2. Trade-off between overall prediction accuracy and individual identification

As shown in Figure S2, during the training process of SwiFUN with MSE loss, the diagonal median and diagonality index initially increase together, but at some point, the diagonal mean starts decreasing

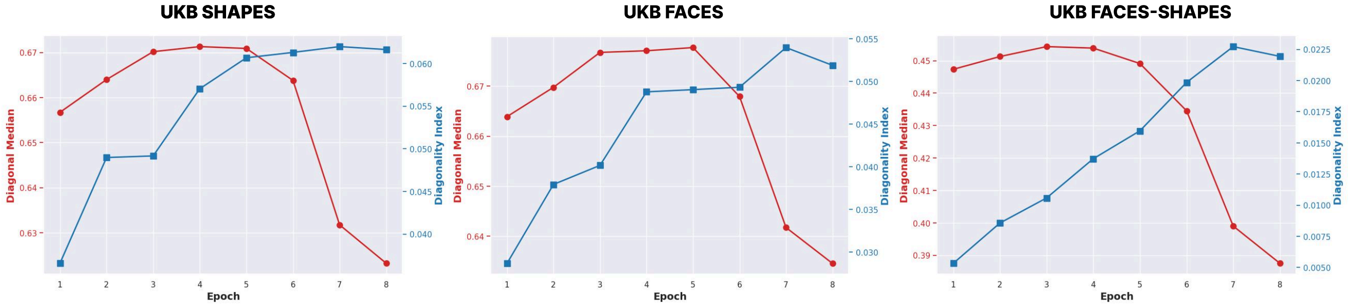

Figure S2: Overall prediction accuracy (diagonal mean) and individual identification (diagonality index) while training SwiFUN (mse)

while the diagonality index continues to increase. This indicates that initially, the model is trained to increase overall prediction accuracy, similar to the group mean activity. However, at a certain point, the model shifts its focus towards capturing subtle individual differences at the expense of overall prediction accuracy. However, there is a drawback regarding the sharp decrease in the diagonal median compared to the increase in the diagonality index. Therefore, in this study, we addressed this issue by incorporating the Reconstruction-Contrastive loss, allowing the model to train in a direction that avoids excessive convergence towards group means and instead reveals individual differences.

3. Performance of ConnTask with different numbers of independent components

Table S1: Performance of ConnTask

Task	IC	Diagonal Median	Diagonality Index	KS_D	Diag. Percent. Mean
SHAPES	21	0.593 ± 0.002	0.062 ± 0.000	0.318 ± 0.015	0.991 ± 0.003
SHAPES	**55**	0.600 ± 0.001	0.069 ± 0.000	0.344 ± 0.013	0.993 ± 0.003
FACES	21	0.604 ± 0.001	0.055 ± 0.000	0.305 ± 0.001	0.989 ± 0.003
FACES	**55**	0.611 ± 0.002	0.061 ± 0.001	0.335 ± 0.002	0.992 ± 0.004
F-S	21	0.371 ± 0.004	0.05 ± 0.001	0.207 ± 0.005	0.943 ± 0.01
F-S	**55**	0.380 ± 0.003	0.056 ± 0.001	0.233 ± 0.006	0.953 ± 0.008

In Table S1, we compared the performances of ConnTask with different independent components(IC) over three emotion contrasts in UKB data. We observed that more number components (55) have a positive effect on both overall prediction accuracy (diagonal median) and individual identification (diagonality index, Effect size D of Kolmogorov-Smirnov test, and Diagonal Percentile Mean) than fewer components (21). Thus, we compared ConTasks, which used 55 independent components, with SwiFUN.

4. The effect of contrastive loss term in Reconstruction-Contrastive loss

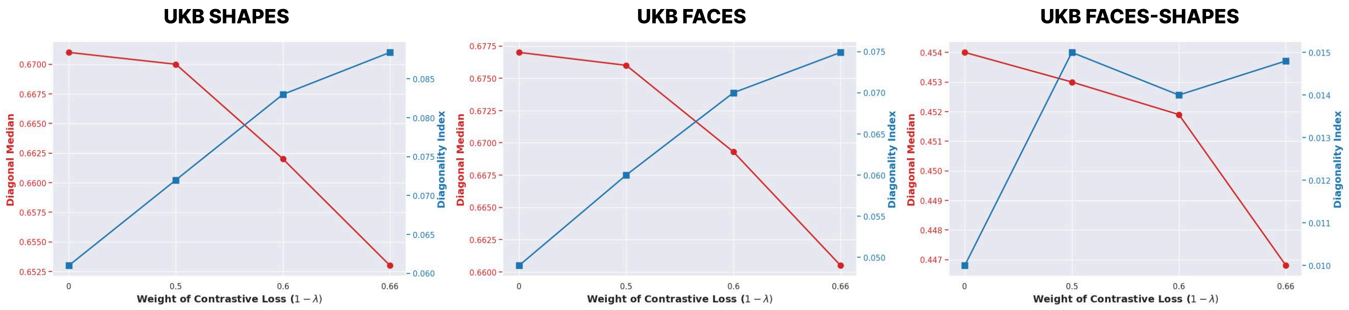

Figure S3: Effect of Contrastive loss term in Reconstruction-Contrastive loss

In Figure S3, we investigated how the diagonal median and diagonality index vary with the adjustment of the weight of the contrastive loss in the RC loss. We conducted experiments with three settings for $1-\lambda$: 0 (L_R only), 0.5 ($L_R : L_C = 1 : 1$), 0.6 ($L_R : L_C = 1 : 1.5$), and 0.66 ($L_R : L_C = 1 : 2$). The results showed that as we increased the weight of the contrastive loss term in all contrast maps, the diagonal median decreased while the diagonality index increased. However, compared with the results of predicting the SHAPES contrast map in Figure S2, where no contrastive loss term was used, we can observe that the increase in the diagonality index is much more significant compared to the relatively small decrease in the diagonal median. For instance, in the case of predicting the SHAPES contrast in Figure S2, after the diagonal median converged at 0.671, it decreased by 0.04, while the diagonality index increased by only 0.001. On the other hand, while $1-\lambda$ increased from 0 to 0.66, the diagonality index significantly improved by 0.027, with a similar decrease in the diagonal median by 0.05. This suggests that utilizing the RC loss can significantly enhance individual identification performance while only minimally compromising overall prediction accuracy.

5. Kolmogorov-Smirnov test for estimating the difference between diagonal and off-diagonal correlations

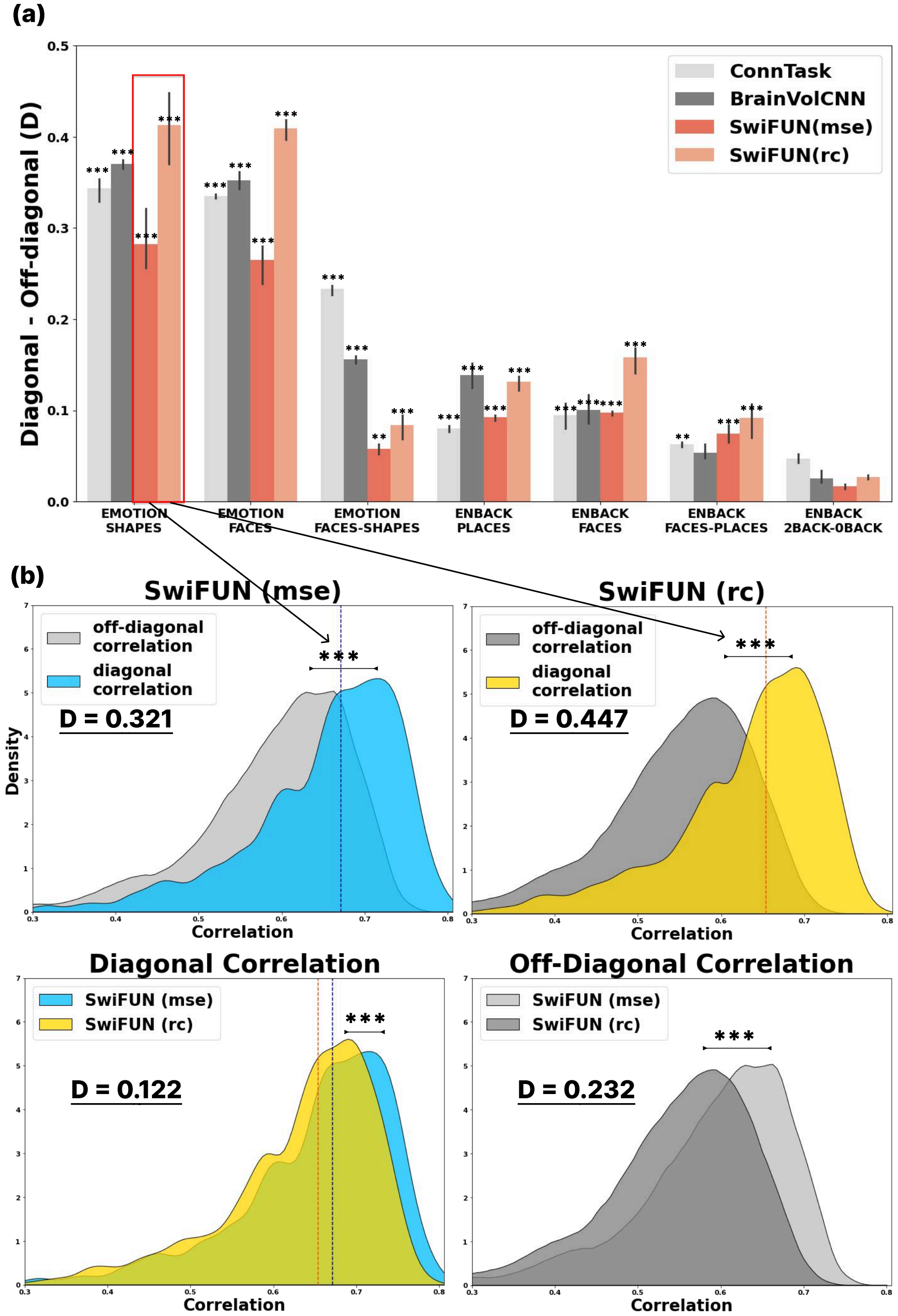

Figure S4: (a) Effect Size and p-value (asterisk over the bar) of the Kolmogorov-Smirnov Test (D) represent the model's performance in capturing individual difference, (b) KDE plots illustrate the diagonal and off-diagonal correlations of the two kinds of SwiFUN in the UKB SHAPES contrast. The dark blue and orange vertical lines mean the median of diagonal correlations of SwiFUN (mse) and SwiFUN (rc), respectively.

6. Negative Correlation between Head Motion Level of fMRI Scans and ConnTask's Prediction Performance

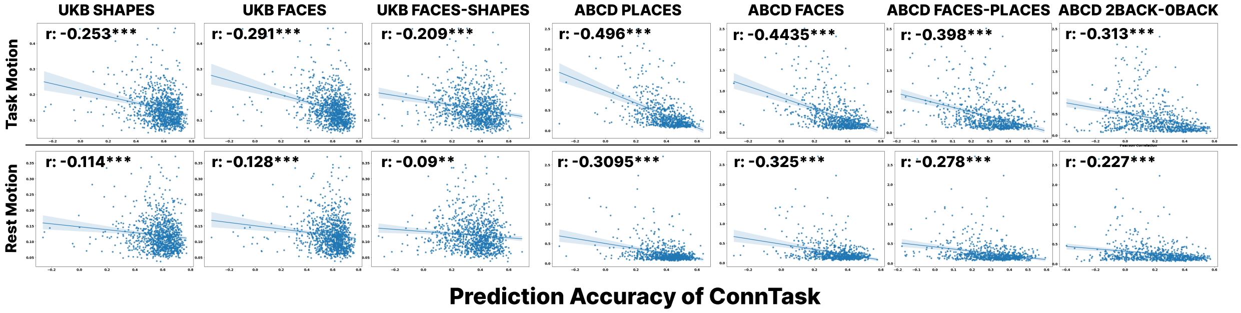

Figure S5: Scatter plots showing a negative correlation between mean head motion and prediction accuracy of ConnTask. Each row represents the averaged head motion of task-based fMRI and resting-state fMRI.

7. Negative Correlation between Head Motion Level of fMRI Scans and Similarity between the Actual Map and Group Average Map

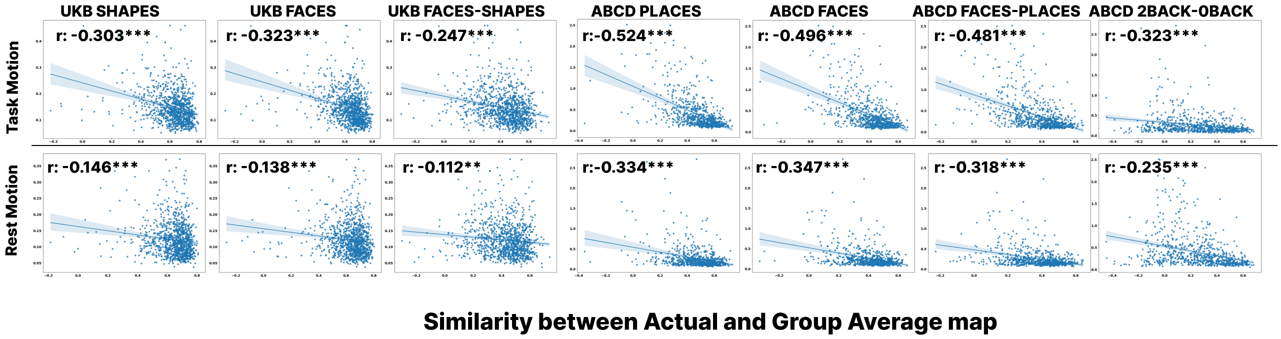

Figure S6: Scatter plots showing a negative correlation between mean head motion and group average map. Each row represents the averaged head motion of task-based fMRI and resting-state fMRI.

8. Additional measures for metadata prediction

Table S2: Additional measures for evaluating task activation maps in predicting individual traits (sex, age, depression, and neuroticism). Each number represents the mean and standard deviation of 20 predictions.

**UKB Shapes**						
		**Real**	**ConnTask**	**BrainVolCNN**	**SwiFUN (mse)**	**SwiFUN (rc)**
**Sex**	ACC	0.78 ± 0.03	0.79 ± 0.02	0.87 ± 0.02	**0.93 ± 0.02**	0.92 ± 0.02
**Age**	Prediction R^2	0.31 ± 0.05	0.21 ± 0.03	0.33 ± 0.04	**0.44 ± 0.04**	0.43 ± 0.04
	MSE	39.40 ± 3.09	44.96 ± 2.81	37.69 ± 3.16	**31.94 ± 2.69**	32.33 ± 2.54
**PHQ-9**	Prediction R^2	-0.16 ± 0.12	-0.12 ± 0.10	-0.25 ± 0.11	-0.13 ± 0.11	**-0.11 ± 0.11**
	MSE	15.87 ± 2.73	15.51 ± 3.22	18.27 ± 2.76	15.62 ± 3.22	**15.31 ± 2.97**
**N-12**	Prediction R^2	-0.18 ± 0.09	**-0.09 ± 0.08**	-0.34 ± 0.18	-0.12 ± 0.12	-0.17 ± 0.16
	MSE	12.98 ± 1.29	**12.03 ± 1.50**	14.70 ± 2.09	12.25 ± 1.40	12.75 ± 1.57
**UKB Faces**						
		**Real**	**ConnTask**	**BrainVolCNN**	**SwiFUN (mse)**	**SwiFUN (rc)**
**Sex**	ACC	0.78 ± 0.02	0.80 ± 0.02	0.88 ± 0.02	**0.92 ± 0.02**	0.92 ± 0.02
**Age**	Prediction R^2	0.30 ± 0.05	0.20 ± 0.03	0.34 ± 0.05	0.41 ± 0.04	**0.44 ± 0.04**
	MSE	40.01 ± 3.33	45.76 ± 3.43	37.06 ± 3.16	33.28 ± 2.58	**31.76 ± 2.49**
**PHQ-9**	Prediction R^2	-0.17 ± 0.14	**-0.13 ± 0.10**	-0.22 ± 0.10	-0.16 ± 0.11	-0.15 ± 0.12
	MSE	16.03 ± 2.85	**15.66 ± 3.17**	17.92 ± 3.43	16.01 ± 3.34	15.82 ± 3.04
**N-12**	Prediction R^2	-0.20 ± 0.08	**-0.09 ± 0.06**	-0.41 ± 0.21	-0.16 ± 0.13	-0.16 ± 0.14
	MSE	13.19 ± 1.56	**11.99 ± 1.51**	15.40 ± 1.94	12.72 ± 1.54	12.68 ± 1.61
**UKB Faces-Shapes**						
		**Real**	**ConnTask**	**BrainVolCNN**	**SwiFUN (mse)**	**SwiFUN (rc)**
**Sex**	ACC	0.70 ± 0.03	0.77 ± 0.02	0.82 ± 0.02	**0.91 ± 0.02**	0.91 ± 0.02
**Age**	Prediction R^2	0.10 ± 0.06	0.12 ± 0.05	0.37 ± 0.06	0.41 ± 0.04	**0.41 ± 0.05**
	MSE	51.05 ± 4.21	50.21 ± 4.65	35.29 ± 3.33	33.54 ± 2.61	**33.26 ± 2.59**
**PHQ-9**	Prediction R^2	-0.24 ± 0.15	**-0.17 ± 0.05**	-0.20 ± 0.10	-0.25 ± 0.13	-0.22 ± 0.11
	MSE	17.10 ± 3.29	**16.27 ± 3.70**	17.58 ± 3.35	17.12 ± 3.15	16.89 ± 3.30
**N-12**	Prediction R^2	-0.25 ± 0.12	**-0.19 ± 0.08**	-0.38 ± 0.17	-0.25 ± 0.15	-0.26 ± 0.14
	MSE	13.75 ± 2.14	**13.08 ± 1.77**	15.18 ± 2.15	13.63 ± 1.52	13.83 ± 1.74

For sex and age predictions, the task activation maps predicted by SwiFUN models demonstrated significantly higher accuracy, R², and Mean Squared Error (MSE) performance across all contrasts compared to both real activation maps and those predicted by other baseline models ($p < 0.001$). For predictions of depressive symptoms (PHQ-9) and neuroticism (N-12), the R^2 values were negative, which indicated

low model fit and complicated direct comparisons. In these cases, SwiFUN models generally showed slightly better R^2 scores and mean square errors than real activation maps and similar or slightly lower performance relative to ConnTask. However, the differences in MSE and R^2 among models for these tasks were not statistically significant.
